# Sexual Dimorphic Metabolic Alterations in Hepatitis C Virus-infected Patients: A Community-Based Study in a Hepatitis B/Hepatitis C Virus Hyperendemic Area

**DOI:** 10.1097/MD.0000000000003546

**Published:** 2016-05-06

**Authors:** Jing-Hong Hu, Mei-Yen Chen, Chau-Ting Yeh, Huang-Shen Lin, Ming-Shyan Lin, Tung-Jung Huang, Ming-Ling Chang

**Affiliations:** From the Department of Gastroenterology and Hepatology, Department of Internal Medicine, Chang Gung Memorial Hospital, Yunlin, Taiwan (J-HH); College of Nursing, Chang Gung University of Science and Technology, Putz City, Chiayi County, Taiwan (M-YC); Liver Research Center and Division of Hepatology, Department of Gastroenterology and Hepatology, Chang Gung Memorial Hospital, Linkou, Taiwan (C-TY, M-LC); Division of Infection Disease, Department of Medicine, Chang Gung Memory Hospital, Chia-yi, Taiwan (H-SL); Division of Cardiology, Chang-Gung Memorial Hospital, Yunlin, Taiwan (M-SL); Division of Thoracic Medicine, Department of Internal Medicine, Chang Gung Memorial Hospital, Yunlin, Taiwan (T-JH); and Department of Medicine, College of Medicine, Chang Gung University, Taoyuan, Taiwan, (M-LC).

## Abstract

The impact of sex on metabolic alterations in individuals with hepatitis B virus (HBV) or hepatitis C virus (HCV) infection remains elusive.

A community-based study was performed to assess sex, age, body mass index, the lipid profile, blood pressure, glucose, alanine aminotransferase, HBV surface antigen (HBsAg), and HCV antibody levels, smoking and alcohol drinking habits, and cardiometabolic diseases, including diabetes, hypertension, cardiovascular events, and renal diseases. The HCV-RNA level and genotype were further assessed in HCV antibody-positive subjects, and the hepatitis B e antigen and HBV-DNA levels were further examined in HBsAg-positive subjects.

Among the 10,959 adults enrolled, 1949 (17.8%) and 1536 (14.0%) were HBV and HCV-infected, respectively. Univariate and multivariate analyses showed that the lipid profile and hypertension were independently associated with HCV infection (95% confidence intervals of odds ratios [OR 95% CI]: total cholesterol [TC] = 0.508–0.677; triglycerides = 0.496–0.728; hypertension = 0.669–0.937), but not with HBV infection. Consistently, HCV, but not HBV infection, was negatively associated with the TC and triglyceride levels (OR 95% CI for TC: 0.450–0.601; triglycerides: 0.443–0.671). Generalized linear models revealed that HCV infection, sex, and age interactively affected the lipid profile (OR 95% CI TC = 1.189–1.385; triglycerides = 1.172–5.289). Age-stratification analysis showed that the lipid levels were lower in both the HCV-positive females aged ≥49 years (TC, *P* < 0.001; triglycerides, *P* = 0.001) and males of all ages (TC, *P* < 0.001; triglycerides, *P* < 0.001) compared with their sex and age-matched HCV-negative counterparts. HCV infection was associated with a higher body mass index (≥49 years, *β* = 0.405, *P* = 0.002) and increased rates of cardiovascular events (<49 years, OR 95% CI 1.23–9.566), diabetes (≥49 years, OR 95% CI 1.114–1.932), and renal diseases (≥49 years, OR 95% CI 1.23–9.55), and with a lower rate of hypertension (≥49 years, OR 95% CI 0.616–0.964) in the females, but not in the males, as determined by multivariate analyses.

Only HCV infection was associated with metabolic alterations in this HBV/HCV-hyperendemic area. Females aged ≥49 years and males of all ages exhibited HCV-associated hypolipidemia. HCV-associated cardiometabolic diseases were evident only in the females. Sex dimorphism in HCV-associated metabolic complications warrants personalized follow-up of HCV-positive patients.

## Abstract 2

Supplemental Digital Content is available in the text

## INTRODUCTION

Hepatitis C virus (HCV) and hepatitis B virus (HBV) infect an estimated 185 and 350 million individuals worldwide, respectively,^1^ and they are both important human pathogens responsible for acute and chronic liver diseases. In addition to cirrhosis and hepatocellular carcinoma, HCV is thought to cause metabolic alterations, resulting in hypolipidemia, hepatic steatosis, insulin resistance (IR), metabolic syndrome (MS), and diabetes.^2,3^ In particular, much of the HCV life cycle, including naïve cell entry, RNA replication, assembly, and secretion, is closely linked to the host lipid metabolism.^2^ Chronic HBV infection is associated with hypolipidemia, especially hypotriglyceridemia,^4^ but not with hepatic steatosis, IR, or diabetes.^5^ However, conflicting data on HBV-associated hypolipidemia have been reported,^6^ and no direct link between the HBV life cycle and host metabolism has been noted to date. Furthermore, in contrast with HCV, the eradication of chronic HBV infection remains a remote goal;^7^ the data on HBV-associated metabolic alterations are mainly based on case-control studies rather than on cohort studies evaluating the influence of viral clearance.^3^ Therefore, the precise effects of HBV infection on host metabolism remain elusive.

In humans, sex-specific specialization is associated with distinct body fat distribution and energy substrate utilization patterns, which are mainly influenced by the menstrual phase in females.^8^ The risk of cardiometabolic diseases, including cardiovascular events, hypertension, diabetes, and renal diseases, is lower in premenopausal women than in age-matched men.^9^ However, within 10 years of menopause, the risk in women increases to a level similar to that in men. Estrogen deficiency-related alterations in lipid metabolism may be crucial to this increase.^10^ Specifically, a reduction in the high-density lipoprotein cholesterol (HDL-C) level and increases in the total cholesterol (TC), triglycerides (TGs), fibrinogen, and lipoprotein levels have been observed after menopause.^11^ Chronic HCV infection significantly increases the risk of cardiovascular events,^12^ in sharp contrast with chronic HBV infection, which does not seem to affect this risk.^13^ It remains undetermined whether sex dimorphism influences HCV or HBV-associated metabolic alterations or cardiometabolic diseases.

In Taiwan, the prevalence of HBV infection is as high as 17.3%, as the vertical transmission rate was elevated in the past due to lack of availability of an HBV vaccine.^14^ The prevalence of HCV infection has been determined to be 1% to 3% in Taiwan, whereas it is as high as 6% to 30% in southern Taiwan specifically.^15^ This high prevalence is primarily attributed to infection through iatrogenic routes due to local individuals’ medical-seeking behaviors, for example, requesting to receive frequent intravenous injections for minor diseases,^16^ and the use of folk remedies, such as acupuncture and cutting of the skin with nonsterilized knives, as observed in many HCV-endemic areas.^16–19^ Clarification of the effects of sex on metabolic alterations and cardiometabolic diseases in individuals infected with HBV or HCV is crucial for public health promotion. Therefore, we attempted to elucidate these associations by analyzing community-based screening data adjusted for the demographic, metabolic, and liver profiles of patients from Mailiao, Taiwan—an area hyperendemic for HBV and HCV infections.

## METHODS

### Community Health Screening and Follow-Up

Between September 2012 and August 2013, township-wide community health screening of the residents of Mailiao Township, Yunlin County, was conducted. All of the residents were invited by mail, telephone, and the media to undergo comprehensive health examination. A total of 12,348 participants received this examination. Serum HCV-RNA and HCV genotypes were further assessed in HCV antibody (Ab)-positive individuals, and the hepatitis B e antigen (HBeAg) and HBV-DNA levels were further examined in HBV surface antigen (HBsAg)-positive subjects within 3 months after the health examination. We excluded subjects with symptoms and signs of acute infection, hemochromatosis, Wilson disease, autoimmune hepatitis, primary biliary cholangitis, primary biliary sclerosis, or malignancy; those who had received anti-HBV or anti-HCV therapy within the past 6 months; those aged <18 years of age; those with missing screening data; and those who tested positive for HCV Ab, but negative for HCV-RNA. Finally, 10,959 subjects were enrolled and analyzed, including 4686 males and 6273 females. The body weights and heights of these subjects were measured, and their body mass indices (BMIs) were calculated as weight in kg divided by height in m^2^. Venous blood was drawn from all the subjects under fasting conditions, and blood pressure was measured under standard conditions during the day. The estimated glomerular filtration rate (eGFR) was calculated according to the Cockcroft–Gault equation. Smoking and alcohol drinking habits (alcohol consumption ≥10 g/day for women and 20 g/day for men)^20^ were identified using a questionnaire. Abdominal ultrasound was performed on each patient to detect fatty liver and cirrhosis. Diagnosis of liver cirrhosis was supplemented with clinical features such as esophageal or gastric varices and thrombocytopenia, as described elsewhere.^21^

### Metabolic Syndrome and Cardiometabolic Diseases

Metabolic syndrome scores were calculated according to the criteria of the National Cholesterol Education Program's Adult Treatment Panel III.^22^ The Caucasian central obesity criteria were replaced with modified criteria proposed by the Taiwan Health Promotion Administration of the Ministry of Health and Welfare in 2007, including waist circumferences of ≥90 cm for men and ≥80 cm for women (BMI >27 kg/m^2^). Enrolled subjects with a past history of cardiometabolic diseases, including cardiovascular events (eg, myocardial infarction, ischemic stroke, or coronary revascularization), diabetes, hypertension, or renal disease, were identified using the International Classification of Diseases, Ninth Revision, Clinical Modification, by patient reports, and confirmed by a review of medical records/registries.

### Biochemistry

The lipid profile (TC, TGs, and HDL-C) and alanine aminotransaminase (ALT), fasting glucose (Glu), HBsAg, HBeAg (Abbott Laboratories, North Chicago, IL), HBV-DNA (Digene Corp., Gaithersburg, MD), HCV Ab (Abbott Laboratories, Chicago, IL), and HCV-RNA levels (Roche Diagnostics, Tokyo, Japan) and HCV genotypes (Roche Diagnostics) were assessed at the clinical pathology or liver research laboratory of the authors’ affiliated hospital using routine automated techniques.

### Statistical Analysis

Statistical Product and Service Solutions (SPSS) version 21 (SPSS, Inc., Chicago, IL) was used for all data management and analyses. Continuous variables are presented as the mean ± standard deviation (SD), and categorical variables are presented as frequencies and percentages. Analysis of variance (ANOVA) and post hoc tests were performed to compare the different variables among ≥3 groups. For comparisons between 2 groups, continuous variables were analyzed using Student *t* test, and categorical variables were analyzed using the chi-square test or Fisher exact test as appropriate. Univariate regression models were used to assess the relationships of the independent variables with the dependent variables; independent variables found to be associated with dependent variables were included in multivariate regression models. The collinearities among the different variables were detected by linear regression tests. The effects of modifications of the variables were evaluated with generalized linear models (GLMs) as indicated. Statistical significance was defined at the 5% level based on a 2-tailed test of the null hypothesis.

### Institutional Review Board

The study protocol conformed to the ethical guidelines of the 1975 Declaration of Helsinki and was approved by the institutional review board of the Chang Gung Memorial Hospital. All the participants provided written informed consent to participate in this study.

## RESULTS

### Baseline Characteristics

The characteristics of the enrolled patients are listed in Table 1. Among the 10,959 patients, 1949 (17.8%) and 1536 (14.0%) were infected with HBV (HBsAg-positive) and HCV (both HCV Ab-positive and HCV-RNA-positive), respectively. The median age of these 10,959 subjects was 44.0 years (range 18.0–102 years), and the mean ages of the male and female subjects were similar. Notably, the males had significantly higher levels of most of the tested parameters than the females, with the exception of the HCV infection prevalence and TC and HDL-C levels, which were higher in the females, and the prevalences of cardiovascular events and renal diseases, which did not differ between the males and females. Two hundred seventy-five (2.5%) of the enrolled subjects were coinfected with both HBV and HCV (Supplementary Table 1). In ANOVA and post hoc analyses, the main differences in metabolic alterations were observed in the comparisons between coinfected patients (group 3) and those with HBV single infection (group 1), and also in those between coinfected patients and those without HBV or HCV infection (group 4), but not in those between coinfected patients and those with HCV single infection (group 2), as shown in Supplementary Table 1.

**TABLE 1:**
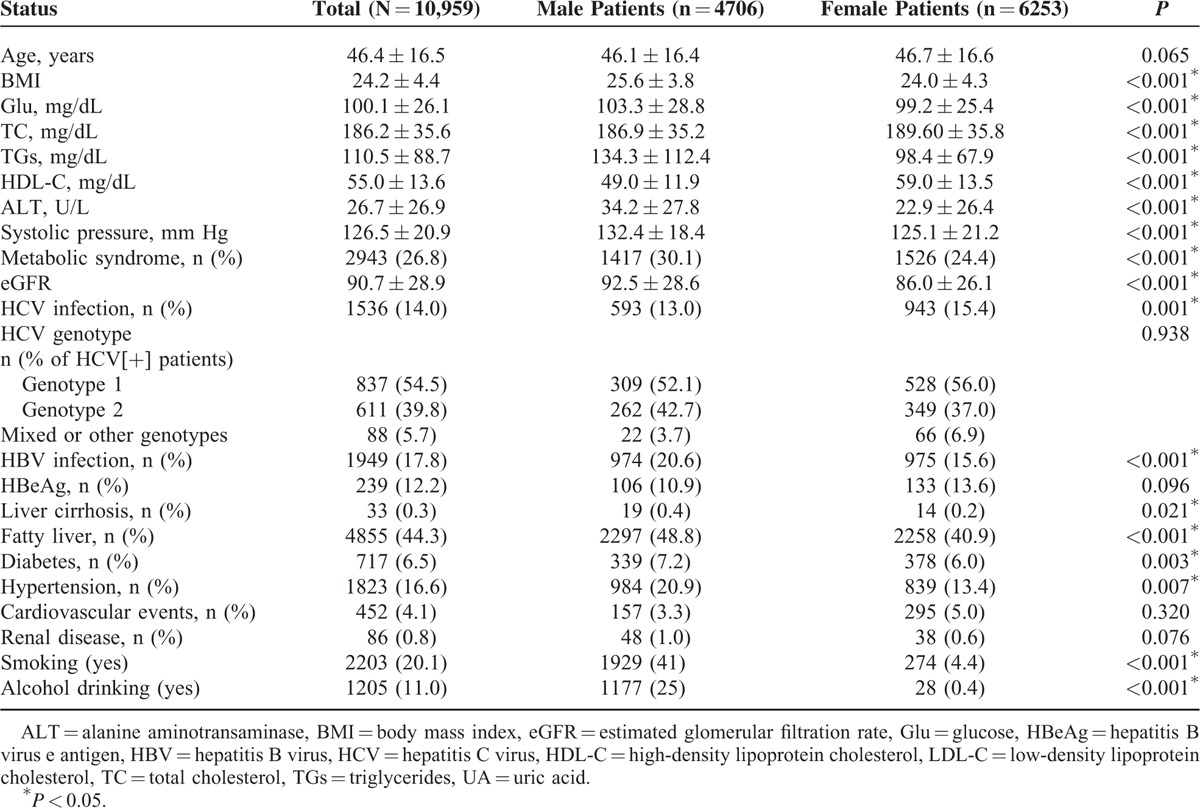
Characteristics of the Enrolled Subjects

### Lipid Profile and Hypertension Were Associated With HCV But Not HBV Infection

The factors associated with HBV and HCV infections are listed in Table 2. Male sex, old age, a high ALT level, and liver cirrhosis were independently associated with HBV infection. Alternatively, female sex, old age, a high ALT level and low TGs, and TC and HDL-C levels were independently associated with HCV infection. Interestingly, hypertension and HCV infection exhibited a positive association in univariate analysis, but displayed a negative association in multivariate analysis. Moreover, HCV, but not HBV infection, was an independent risk factor for low TC and TGs levels (Supplementary Tables 2 and 3) after adjustments for other cofactors, including sex, age, BMI, HDL-C, Glu, ALT, systolic blood pressure, eGFR, MS, liver cirrhosis, fatty liver, diabetes, hypertension, cardiovascular events, renal diseases, and smoking and alcohol drinking habits (TC, odds ratio [OR] 95% confidence interval [95% CI] 0.45–0.601; TGs, OR 95% CI 0.443–0.671).

**TABLE 2:**
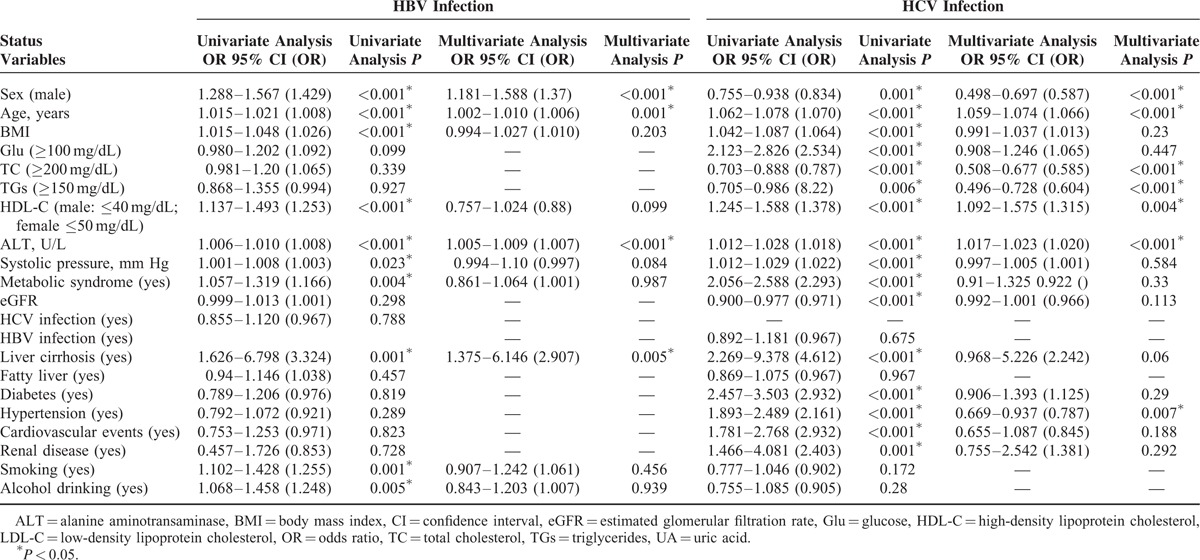
Univariate and Multivariate Logistic Regression Analyses of the Factors Associated With Hepatitis B Virus (HBV) and Hepatitis C Virus (HCV) Infections

The multivariate analysis showed that only the HCV genotype was associated with HCV-RNA levels in HCV-infected patients (HCV genotype 1, 95% CI of β: 2.886–5.78, *P* < 0.001) among factors including sex, age, BMI, Glu, TGs, HDL-C, ALT, systolic blood pressure, eGFR, MS, liver cirrhosis, fatty liver, diabetes, hypertension, cardiovascular events, renal disease, HCV genotype, and the smoking and alcohol drinking habits. The patients infected with HCV genotype 1 had a significantly higher HCV-RNA level than those infected with HCV genotype 2 (6.35 ± 9.23 vs 2.76 ± 4.21 × 10^6^ IU/mL; *P* < 0.001). However, no differences in any of the tested metabolic parameters were noted between the patients infected with HCV genotypes 1 and 2. Subgroup analyses revealed no obvious differences in the metabolic profiles of the HBV patients with and without HBeAg positivity. Further, univariate and multivariate analyses failed to demonstrate an association between the HBV-DNA level and metabolic profile.

### HCV Infection, Sex, and Age Interactively Affected Lipid Profile

Menopause typically begins between 48 and 50 years of age in Taiwanese women^23^; thus, a cut-off age of 49 years, which is considered the landmark age for menopause in Taiwan^24^ and has resulted in the lowest *P* values in determination of the TC and TG levels,^25^ was used in subsequent analyses of the effects of menopause on the lipid profile. The effects of sex, age, HCV infection, and the interactions among these factors on elevations in the TC (≥200 mg/dL) and TGs (≥150 mg/dL) levels, as determined by GLM analysis, are presented in Table 3. HCV infection, sex, and age interactively affected the lipid profile. Among females aged <49 years, the HCV-positive patients had higher ORs for both TC ≥200 mg/dL (OR 95% CI 1.189–1.385) and TGs ≥150 mg/dL (OR 95% CI 1.172–5.289) than the HCV-negative patients.

**TABLE 3:**
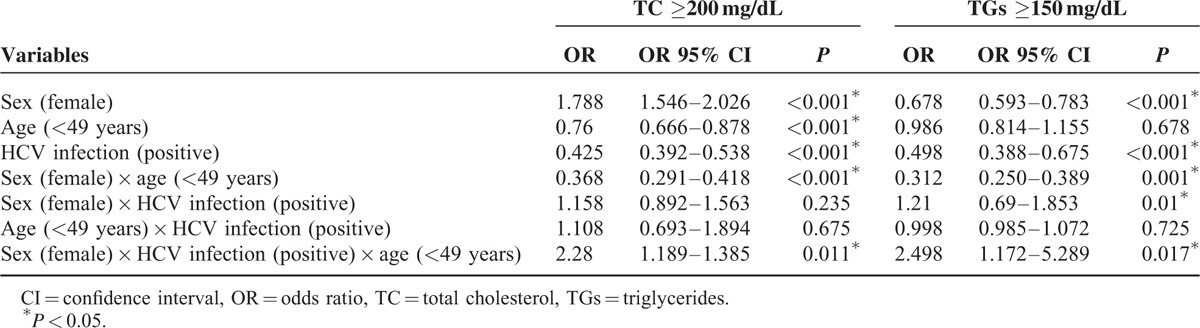
Effects of Sex, Age and Hepatitis C Virus (HCV) Infection and Their Interactions on Cholesterol and Triglyceride Levels

### HCV-positive Males of all Ages and HCV***-***positive Females Aged ≥49 Years Had Lower Lipid Levels Than Their Sex and Age-matched HCV-negative Counterparts

**FIGURE 1:**
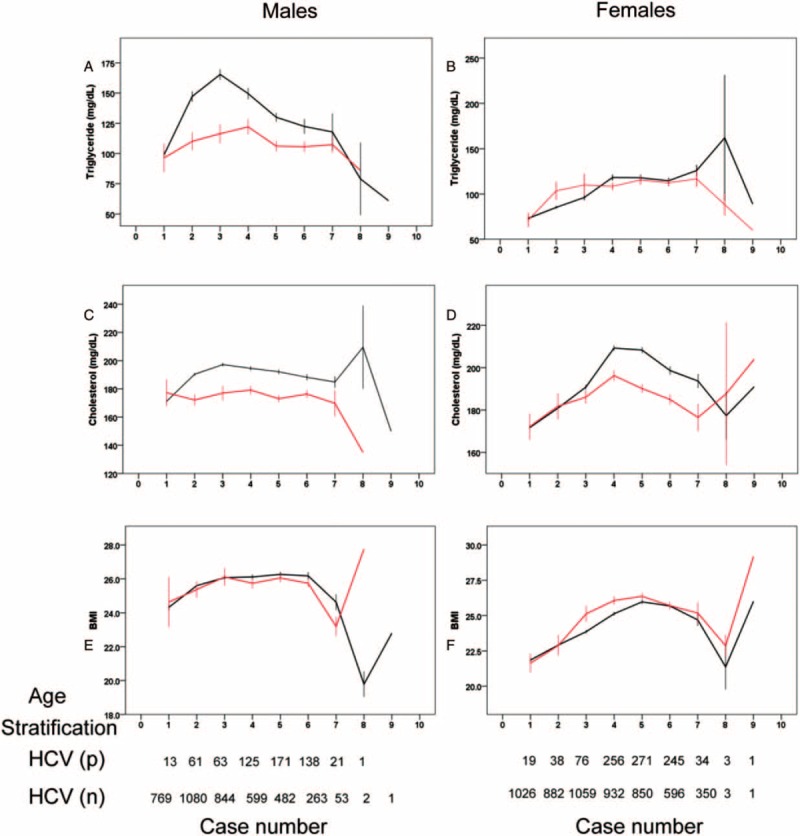
The mean ± standard error of the metabolic parameters, including the triglycerides (A and B), and total cholesterol levels (C and D) and BMIs (E and F), of the male (left panels) and female (right panels) patients, who were positive (red lines) and negative (black lines) for HCV infection. The subjects were stratified by age. Age stratifications: 1: 18.0 to 29.0 years; 2: 29.1 to 39.0 years; 3: 39.1 to 49.0 years; 4: 49.1 to 59.0 years; 5: 59.1 to 69.0 years; 6: 69.1 to 79.0 years; 7: 79.1 to 89.0 years; 8: 89.1 to 99.0 years; and 9: ≥99.1 years. BMI = body mass index, HCV = hepatitis C virus.

Figure 1 shows the mean ± standard error TG and TC levels and BMIs of the sex and age (with each age group spanning 10 years)-matched HCV-positive and negative subjects. Table 4 presents the *P* values, ORs, and 95% CIs of the ORs for the factors associated with HCV infection, as determined after stratification by sex and age (cut-off 49 years) with adjustments for confounders. A marked tendency toward hypolipidemia was observed among the HCV-positive males, who had lower TG and TC levels than the HCV-negative males of all ages (Figure 1A and C, Table 4). However, lower lipid levels were primarily observed in the HCV-positive females aged ≥49 years compared with the HCV-negative females aged ≥49 years. Moreover, the TC and TG levels did not differ between the HCV-positive females aged <49 years and their HCV-negative counterparts (Figure 1B and D, Table 4). No significant difference in BMI was noted between the HCV-positive and negative males in any of the age groups (Figure 1E, Table 4). In contrast, the HCV-positive females aged ≥49 years had a higher BMI than the HCV-negative females aged ≥49 years (Figure 1F, Table 4). Further, HCV infection was consistently associated with a higher BMI in the females aged ≥49 years (*β* = 0.405, *P* = 0.02) when BMI was used as a dependent factor. To elucidate the patterns of metabolic alterations in the patients coinfected with HBV and HCV, subgroup analysis were performed with further stratification of the subjects into the following 4 groups: HBV single infection (group 1), HCV single infection (group 2), HBV and HCV coinfection (group 3), and neither HBV nor HCV infection (group 4) (Supplementary Figure 1). The results revealed differences in the lipid profiles and BMIs mainly between groups 1 and 3, groups 3 and 4, and groups 2 and 4. Moreover, the patterns of HCV-associated sexual dimorphism in the lipid and BMI alterations (Figure 1) were maintained in the group 3 subjects (Supplementary Figure 1). The group 2 and group 3 subjects had similar lipid profiles and BMIs.

**TABLE 4:**
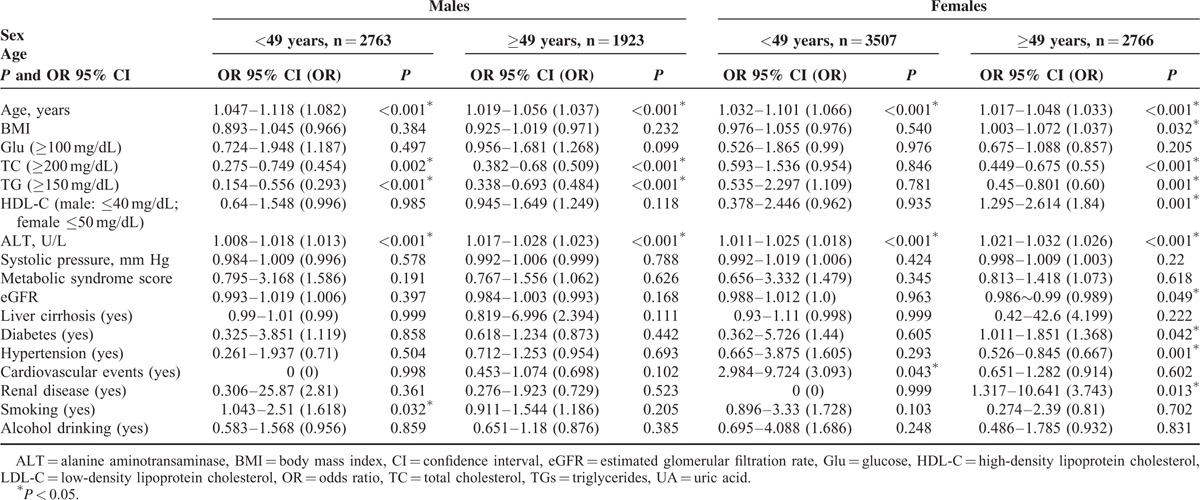
Multivariate Logistic Regression Analyses of the Factors Associated With Hepatitis C Virus (HCV) Infections Stratified by Sex and Age (Cut-off 49 Years)

### Associations Between Cardiometabolic Diseases and HCV Infection Were Only Evident in Females

As shown in Table 4, no association between HCV infection and any cardiometabolic disease was noted among the males. In contrast, HCV infection was positively associated with cardiovascular events in females aged <49 years, and it was positively associated with diabetes and renal disease, and negatively associated with hypertension in those aged ≥49 years. These relationships were further confirmed using the cardiometabolic diseases as dependent factors, which revealed consistent associations of HCV infection with cardiovascular events in females aged <49 years (OR 95% CI 1.23–9.566) and with diabetes (OR 95% CI 1.114–1.932), renal diseases (OR 95% CI 1.23–9.55) and hypertension in those aged ≥49 years (OR 95% CI 0.616–0.964).

## DISCUSSION

To the best of our knowledge, the current study is the first to comprehensively examine HCV-associated sexual dimorphic metabolic alterations in an HBV and HCV-hyperendemic area. The most compelling results were as follows: HCV but not HBV infection was independently associated with lipid alterations; sex, age, and HCV infection interactively affected the lipid profile; lower lipid levels were observed in the HCV-positive males of all ages and in the HCV-positive females aged ≥49 years compared with their age-matched HCV-negative counterparts; however, only the HCV-positive females aged ≥49 years had a higher BMI than their counterparts; and after stratification by sex and age, HCV infection was positively associated with diabetes, cardiovascular events, and renal diseases, and negatively associated with hypertension in females, but not in males.

Previous studies of the relationship between the TG level and HBV infection have been small or medium-scale studies^5,26^ conducted on subgroups with elevated ALT levels,^4^ or large-scale studies with limited adjustments for confounders.^27^ After comprehensive adjustments for all of the crucial demographic, metabolic, and liver parameters of the 10,959 enrolled subjects, a negligible association between HBV infection and the metabolic profile was confirmed by multivariate analyses. Subgroup analysis further confirmed that HCV infection was the main cause of alterations in the lipid levels and BMIs in the HBV and HCV coinfected subjects. This information is extremely important for HBV/HCV-hyperendemic areas. The close association detected between HCV infection and metabolic alterations, particularly hypolipidemia, is compatible with previous results.^3,12,28^ The favorable effects of estrogen on the lipid profile likely protected the females aged <49 years from HCV-associated hypolipidemia, which might resurface after 49 years of age due to estrogen decline after menopause.^11^ A study based on 2 independent National Health and Nutrition Examination Survey cohorts revealed similar results, showing that age affects HCV-associated hypolipidemia in females, but not in males.^29^ Furthermore, the prevalence of visceral adiposity is elevated in HCV-positive compared with HCV-negative individuals.^30^ Interestingly, in the current study, the HCV-positive females aged ≥49 years tended to have a higher BMI than their HCV-negative counterparts, whereas this phenomenon was not evident among the males. There are several well-known sexual dimorphic metabolic alterations.^31,32^ In patients with HCV infection, lipogenesis is elevated, whereas cholesterol synthesis is impaired.^33^ Further evaluation is required to determine whether the enhanced lipogenesis and decreased cholesterol synthesis prominent in HCV-infected females and males, respectively, are responsible for the sexual dimorphic HCV-associated alterations in the BMI and lipid profile.

Despite the favorable lipid profile associated with HCV infection, the HCV-infected patients are at an increased risk of cardiometabolic diseases.^34^ This risk is mainly regarded as link to deteriorated glucose metabolism.^35^ In the current study, an association between cardiometabolic diseases and HCV infection was only evident among the females; their increased BMI and delayed onset of hypolipidemia both potentially contributed to this relationship. Furthermore, the positive associations between diabetes/renal diseases and HCV infection observed in the females aged ≥49 years indicate that these diseases may be late-onset HCV-associated complications that mainly occur in older patients, in contrast with cardiovascular events. HCV infection affects men and women differently.^36^ Chronically infected women are more likely to exhibit spontaneous HCV clearance and are less likely to display disease progression. However, the rate of disease progression changes over time in women and is directly related to the reproductive status.^37^ As women age, they are at an increased risk of HCV-associated complications.^38^ Thus, the increased prevalence of HCV infection among the females compared with the males (Table 1), particularly among those aged 50 to 80 years (Figure 1), might indicate that the females suffered from severe or recurrent HCV infections that did not spontaneously clear and promoted the occurrence of cardiometabolic complications. Whether medical-seeking behaviors^16–19^ led to the more severe HCV infections in these individuals than expected demands future investigation. Moreover, the male sex might be much more important than HCV infection as a confounder in estimation of the risks of cardiometabolic diseases.^39^ Further, the association between HCV infection and smoking (Table 4), a well-known factor for cardiovascular events,^40^ in the male subjects aged <49 years, might have masked the link between HCV infection and cardiovascular events in this subgroup; these would explain why HCV-associated cardiometabolic diseases were not observed in the male-only group. Although a positive association between hypertension and HCV infection has been found in a study of 173 chronic hepatitis C patients^41^ and in the current study by univariate analysis, paradoxically, a negative association was demonstrated by multivariate analysis, particularly among the females aged ≥49 years. Given that hypercholesterolemia is an established risk factor for hypertension,^42^ the delayed hypolipidemia observed in the HCV-positive females aged ≥49 years might have accounted for the protective role of HCV infection against hypertension in this subgroup after adjusting for the other confounders.

A recent study of the northern Taiwanese population, which has a low prevalence of HCV infection (HCV Ab positivity: 2.7%), has revealed that HCV Ab positivity is not associated with MS, irrespective of age and sex.^43^ In contrast, the current study demonstrated the presence of sexual dimorphic metabolic alterations in subjects from the southern Taiwanese population, which has a high prevalence of HCV infection (14.0%). These conflicting results suggest that HCV-associated sexual dimorphism might not be detected unless data mining is performed on a large sample from an HCV-hyperendemic area using sophisticated statistical analyses.

There are some limitations of the current study. First, it is a cross-sectional study. Future longitudinal studies are required to verify the possible causal relationships between chronic HCV infection and sexual dimorphic metabolic alterations. Second, latent infections of human immunodeficiency virus (HIV) (shares the same transmission route with HCV) and cytomegalovirus (CMV), which are both important modifiers of cardiovascular events and the lipid profile,^44,45^ were not assessed. According to the database of the Centers for Disease Control of Taiwan, the prevalence of HIV among the residents of Yunlin County was less than 0.05% in 2013.^46^ Considering this low prevalence, HIV is not a significant confounder of HCV-associated metabolic alterations. An impact of CMV infection is mainly evident in nonobese subjects,^45^ and it may be negligible due to the low prevalence (<5%) of cardiometabolic complications in the subjects with a BMI of <24 in the current study. Third, fatty liver was diagnosed by ultrasonography, which has a sensitivity and specificity of approximately 60% to 94% and 84% to 95%,^47^ respectively, and may result in the under or overdiagnosis of fatty liver. Future study utilizing liver biopsy or magnetic resonance spectroscopy^20^ to confirm fatty liver in HCV-infected subjects might reveal the precise relationship between HCV infection and fatty liver.^48^

In summary, in areas hyperendemic for both HBV and HCV infections, HCV, but not HBV infection, is associated with lipid alterations and cardiometabolic diseases. In addition, sex, age, and HCV infection interactively affect the lipid profile. HCV-associated hypolipidemia was consistently detected in the males of all ages, but was only evident in the females aged ≥49 years. HCV-associated increases in BMI and in the prevalences of cardiovascular events, diabetes, and renal diseases, and also a decrease in the prevalence of hypertension, were observed only in the females. In the present era, in which most HCV infection is eradicable by anti-HCV regimens,^3^ it remains undetermined whether HCV-associated metabolic alterations are completely reversible after viral clearance. A thorough follow-up study examining metabolic alterations and associated diseases among HCV-infected patients, particularly among female patients, is required. Ultimately, personalized care for individuals with HCV-associated diseases may be achieved in the near future.

## Supplementary Material

Supplemental Digital Content
