# Supplementary material for: Sexual Dimorphic Metabolic Alterations in Hepatitis C Virus-infected Patients: A Community-Based Study in a Hepatitis B/Hepatitis C Virus Hyperendemic Area
Source: Medicine (Baltimore). 2016 May 6;95(18):e3546. doi: 10.1097/MD.0000000000003546 (PMC4863783; doi:10.1097/MD.0000000000003546)
Supplement: Supplemental Digital Content [file medi-95-e3546-s001.doc]

**Supplementary Figure 1.** The mean +/- standard error of the metabolic parameters, including the triglycerides (A and B) and total cholesterol levels (C and D) and BMIs (E and F), of the male (left panels) and female (right panels) patients who were positive for HBV infection (Group 1, orange lines), positive for HCV infection (Group 2, blue lines), positive for both HBV and HCV infections (Group 3, red lines) and negative for both HBV and HCV infections (Group 4, black lines). The subjects were stratified by age (cut-off: 49 yr). Age stratifications: **1**: 18.0–29.0 yr; **2**: 29.1–39.0 yr; **3**: 39.1–49.0 yr; **4**: 49.1–59.0 yr; **5**: 59.1–69.0 yr; **6**: 69.1–79.0 yr; **7**: 79.1–89.0 yr; **8**: 89.1–99.0 yr; and **9**: >99.1 yr.


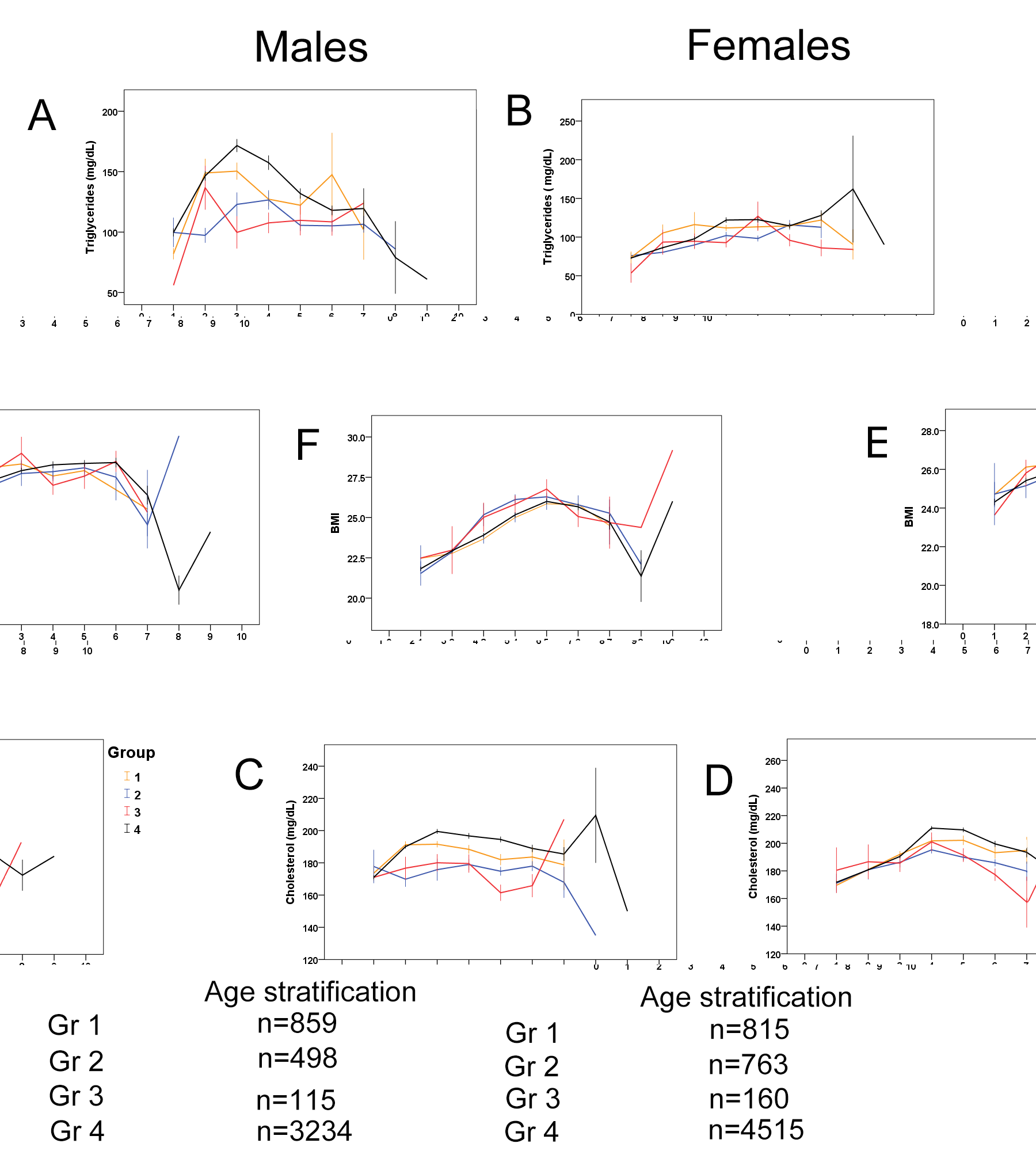


**Supplementary Table 1. The demographics and** metabolic biochemistry of the enrolled subjects.

|  | Group 1 | Group 2 | Group 3 | Group 4 | *p* values of ANOVA  and Post Hoc |
| --- | --- | --- | --- | --- | --- |
| Viral infections | HBV (+)  HCV(-) | HBV (-)  HCV (+) | HBV (+)  HCV (+) | HBV (-)  HCV (-) |  |
| n (%) | 1674 (15.3 %) | 1261(11.5%) | 275(2.5%) | 7749 (70.7%) |  |
| Sex (male) | 859 (51.3%) | 498(37.9%) | 115 (41.8%) | 3234 (41.7%) | ANOVA: *p* <0.001* |
|  |  |  |  |  | Post Hoc tests:  Gr1 vs Gr2: *p* <0.001*  Gr1 vs Gr3: *p=*0.055  Gr1 vs Gr4: *p* <0.001*  Gr2 vs Gr3: *p=*0.629  Gr2 vs Gr4: *p=*0.134  Gr3 vs Gr4: *p=*0.995 |
| Age (yr) | 46.56+/-13.22  44.0(18.0~89.0) | 61.48+/-13.11  63.0(18.0~95.0) | 57.83+/-12.79  59.0(27.0~101.0) | 43.4+/-16.27  40.0(18~102) | ANOVA: *p* <0.001* |
|  |  |  |  |  | Post Hoc tests:  Gr1 vs Gr2: *p* <0.001*  Gr1 vs Gr3: *p* <0.001*  Gr1 vs Gr4: *p* <0.001*  Gr2 vs Gr3: *p=*0.004*  Gr2 vs Gr4: *p* <0.001*  Gr3 vs Gr4: *p* <0.001* |
| BMI | 25.0+/-4.19  24.7(15.2~47.7) | 27.73+/-17.02  25.25(15.1~50.3) | 25.76+/-3.60  25.28(18.637.6) | 24.48+/-4.20  24.12(13.4~47.4) | ANOVA: *p* <0.001* |
|  |  |  |  |  | Post Hoc tests:  Gr1 vs Gr2: *p* <0.001*  Gr1 vs Gr3: *p=*0.053  Gr1 vs Gr4: *p* <0.001*  Gr2 vs Gr3: *p=*0.999  Gr2 vs Gr4: *p* <0.001*  Gr3 vs Gr4: *p* <0.001* |
| Glu (mg/dL) | 100.07+/-24.65  94.6(64~341) | 110.35+/-36.16  100.35(61~517) | 113.14+/-42.94  99.1(70~342) | 99.19+/-24.40  94.0(50428) | ANOVA: *p* <0.001* |
|  |  |  |  |  | Post Hoc tests:  Gr1 vs Gr2: *p* <0.001*  Gr1 vs Gr3: *p* <0.001*  Gr1 vs Gr4: *p=*0.682  Gr2 vs Gr3: *p=*0.469  Gr2 vs Gr4: *p* <0.001*  Gr3 vs Gr4: *p* <0.001* |
| TC (mg/dL) | 189.71+/-33.34  188.0(100~384) | 183.82+/-37.55  182.0(74~442) | 182.80+/-35.34  181.0(98~324) | 189.23+/-35.73  186.0(85~407) | ANOVA: *p* <0.001* |
|  |  |  |  |  | Post Hoc tests:  Gr1 vs Gr2: *p* <0.001*  Gr1 vs Gr3: *p=*0.028*  Gr1 vs Gr4: *p=*0.968  Gr2 vs Gr3: *p=*0.979  Gr2 vs Gr4: *p* <0.001*  Gr3 vs Gr4: *p=*0.031* |
| TG (mg/dL) | 116.16+/-110.09  92.0(29~2569) | 111.65+/-63.99  9.7(30~855) | 106.83+/-74.21  86.5(32~955) | 114.07+/-91.94  90.0(24~1970) | ANOVA: *p* = 0.317 |
|  |  |  |  |  | Post Hoc tests:  Gr1 vs Gr2: *p=*0.614  Gr1 vs Gr3: *p=*0.476  Gr1 vs Gr4: *p=*0.870  Gr2 vs Gr3: *p=*0.886  Gr2 vs Gr4: *p=*0.850  Gr3 vs Gr4: *p=*0.638 |
| HDL-C (mg/dL) | 54.25+/-14.11  53.00(22.0~119.0) | 51.00+/-13.65  49.0(13~135) | 52.11+/-13.93  52.0(21~108) | 55.57+/-13.59  54.0(7~124) | ANOVA: *p* <0.001* |
|  |  |  |  |  | Post Hoc tests:  Gr1 vs Gr2: *p* <0.001*  Gr1 vs Gr3: *p=*0.117  Gr1 vs Gr4: *p=*0.005*  Gr2 vs Gr3: *p=*0.679  Gr2 vs Gr4: *p* <0.001*  Gr3 vs Gr4: *p* <0.001* |
| ALT (U/L) | 32.34+/-28.76  25.00(4~511) | 41.19+/-50.03  29.0(6~1197) | 41.10+/-41.40  29.0(8~326) | 24.01+/-18.88  18.0(3~259) | ANOVA: *p* <0.001* |
|  |  |  |  |  | Post Hoc tests:  Gr1 vs Gr2: *p* <0.001*  Gr1 vs Gr3: *p* <0.001*  Gr1 vs Gr4: *p* <0.001*  Gr2 vs Gr3: *p=*1.0  Gr2 vs Gr4: *p* <0.001*  Gr3 vs Gr4: *p* <0.001* |
| Systolic pressure (mm Hg) | 128+/-19.35  127.0(81~199) | 135.92+/-20.39  137.0(71~206) | 136.66+/-20.88  135.5(80~212) | 126.69+/-20.24  125.00(47~257) | ANOVA: *p* <0.001* |
|  |  |  |  |  | Post Hoc tests:  Gr1 vs Gr2: *p* <0.001*  Gr1 vs Gr3: *p* <0.001*  Gr1 vs Gr4: *p=*0.086  Gr2 vs Gr3: *p=*0.958  Gr2 vs Gr4: *p* <0.001*  Gr3 vs Gr4: *p* <0.001* |
| Metabolic syndrome (n(%)) | 468(28.4%) | 578(43.7%) | 110(39.6%) | 1823(23.9%) | ANOVA: *p* <0.001* |
|  |  |  |  |  | Post Hoc tests:  Gr1 vs Gr2: *p* <0.001*  Gr1 vs Gr3: *p=*0.002*  Gr1 vs Gr4: *p=*0.003*  Gr2 vs Gr3: *p=*0.572  Gr2 vs Gr4: *p* <0.001*  Gr3 vs Gr4: *p* <0.001* |

HBV (+): hepatitis B virus; HCV (+): hepatitis C virus infection; Gr: group; BMI: body mass index; Glu: glucose; TC: total cholesterol; TGs: triglycerides; HDL-C: high-density lipoprotein cholesterol; ALT: alanine aminotransaminase; eGFR: estimated glomerular filtration rate. *, *p*<0.05.

**Supplementary Table 2. Univariate and multivariate logistic regression analyses of the factors associated with the abnormal cholesterol levels (> 200 mg/dL).**

| Variables | Univariate analysis:  95% CI of OR (OR) | Univariate analysis:  *p* value | Multivariate analysis:  95% CI of OR (OR) | Multivariate analysis:  *p* value |
| --- | --- | --- | --- | --- |
| Sex (male) | 0.885~1.037 (0.958) | 0.287 |  |  |
| Age (yr) | 1.019~1.024 (1.021) | <0.001* | 1.013~1.024 (1.018) | <0.001* |
| BMI | 1.059~1.079 (1.069) | <0.001* | 1.032~1.069 (1.05) | <0.001* |
| Glucose ( > 100 ) | 1.442~1.702 (1.567) | <0.001* | 1.173~1.494 (1.324) | <0.001* |
| TG (> 150 mg/dL) | 2.342~2.849 (2.579) | <0.001* | 2.844 ~3.751 (3.266) | <0.001* |
| HDL (male: < 40mg/dL; female < 50) | 0.641~0.767 (0.701) | <0.001* | 0.321~0.423 (0.369) | <0.001* |
| ALT (U/L) | 1.002~1.005 (1.004) | <0.001* | 1.001~1.004 (1.003) | 0.002* |
| eGFR | 0.994~0.997 (0.995) | <0.001* | 0.992~0.998 (0.995) | 0.002* |
| HCV infection (Yes) | 0.701~0.882 (0.787) | <0.001* | 0.45 ~0.601 (0.52) | <0.001* |
| HBV infection (Yes) | 0.961~1.18 (1.065) | 0.74 |  |  |
| Systolic pressure (mm Hg) | 1.014~1.018 (1.016) | <0.001* | 1.006~1.012 (1.009) | <0.001* |
| Metabolic syndrome (Yes) | 1.56~1.855 (1.701) | <0.001* | 0.567~0.769 (0.66) | <0.001* |
| Liver cirrhosis (Yes) | 0.193~1.148 (0.471) | 0.098 |  |  |
| Fatty liver (Yes) | 1.58~1.85 (1.711) | <0.001* | 1.167 ~1.448 (1.300) | <0.001* |
| Diabetes (Yes) | 0.647~0.923 (0.773) | 0.004* | 0.352~0.533 (0.433) | <0.001* |
| Hypertension (Yes) | 1.293~1.632 (1.452) | <0.001* | 0.762~1.014 (0.879) | 0.076 |
| Cardiovascular events (Yes) | 0.705~1.071 (0.869) | 0.187 |  |  |
| Renal disease (Yes) | 0.466~1.32 (0.785) | 0.361 |  |  |
| Smoking (Yes) | 0.871~1.084 (0.907) | 0.607 |  |  |
| Alcohol drinking (Yes) | 0.952~1.237 (1.085) | 0.221 |  |  |

OR: odds ratio; CI: confidence interval. BMI: body mass index; Glu: glucose; UA: uric acid; TC: total cholesterol; TGs: triglycerides; HDL-C: high-density lipoprotein cholesterol; LDL-C: low-density lipoprotein cholesterol; ALT: alanine aminotransaminase; eGFR: estimated glomerular filtration rate; HCV: hepatitis C virus; HBV: hepatitis B virus. *, *p*<0.05.

**Supplementary Table 3. Univariate and multivariate logistic regression analyses of the factors associated with the abnormal triglyceride levels (> 150 mg/dL).**

| Variables | Univariate analysis:  95% CI of OR (OR) | Univariate analysis:  *p* value | Multivariate analysis:  95% CI of OR (OR) | Multivariate analysis:  *p* value |
| --- | --- | --- | --- | --- |
| Sex (male) | 2.316~2.809 (2.563) | <0.001* | 0.96~1.411 (1.164) | 0.122 |
| Age (yr) | 1.011~1.017 (1.014) | <0.001* | 0.979~0.995 (0.987) | 0.001* |
| BMI | 1.159~1.187 (1.173) | <0.001* | 0.954~1.003 (0.978) | 0.089 |
| Glucose (> 100 mg/dL ) | 2.091~2.534 (2.313) | <0.001* | 0.319~0.461 (0.383) | <0.001* |
| TC (>200 mg/dL) | 2.354~2.85 (2.579) | <0.001* | 2.79 ~3.733 (3.227) | <0.001* |
| HDL (male: < 40mg/dL; female < 50) | 4.57~5.577 (5.048) | <0.001* | 2.614~3.752 (3.132) | <0.001* |
| ALT (U/L) | 1.011~1.014 (1.013) | <0.001* | 1.000~1.004 (1.002) | 0.101 |
| eGFR | 1.004~1.007 (1.005) | <0.001* | 0.990~0.999 (0.994) | 0.01* |
| HCV infection (Yes) | 0.715~0.946 (0.822) | 0.006* | 0.443~0.671 (0.545) | <0.001* |
| HBV infection(Yes) | 0.878~1.126 (0.994) | 0.930 |  |  |
| Systolic pressure (mm Hg) | 1.019~1.024 (1.021) | <0.001* | 0.983~0.991 (0.987) | <0.001* |
| Metabolic syndrome (Yes) | 11.532~14.383 (12.879) | <0.001* | 19.78~30.23 (24.45) | <0.001* |
| Liver cirrhosis (Yes) | 0.407~2.438 (0.995) | 0.991 |  |  |
| Fatty liver (Yes) | 3.85~4.747 (4.275) | <0.001* | 1.929~2.46 (2.25) | <0.001* |
| Diabetes (Yes) | 1.454~2.487 (2.089) | <0.001* | 0.926~1.496 (1.17) | 0.183 |
| Hypertension (Yes) | 2.069~2.223 (2.347) | <0.001* | 1.048~1.515 (1.26) | 0.014* |
| Cardiovascular events (Yes) | 0.961~1.527 (1.211) | 0.106 |  |  |
| Renal disease (Yes) | 1.15~3.173 (1.91) | 0.012* | 0.617~2.511 (1.244) | 0.542 |
| Smoking (Yes) | 2.29~2.888 (2.572) | <0.001* | 1.26~1.821 (1.515) | <0.001* |
| Alcohol drinking (Yes) | 2.309~3.029 (2.644) | <0.001* | 1.499~2.26 (1.84) | <0.001* |

OR: odds ratio; CI: confidence interval. BMI: body mass index; Glu: glucose; UA: uric acid; TC: total cholesterol; TGs: triglycerides; HDL-C: high-density lipoprotein cholesterol; LDL-C: low-density lipoprotein cholesterol; ALT: alanine aminotransaminase; eGFR: estimated glomerular filtration rate; HCV: hepatitis C virus; HBV: hepatitis B virus. *, *p*<0.05.
